# The effects of dietary *Spirulina platensis* or curcumin nanoparticles on performance, body chemical composition, blood biochemical, digestive enzyme, antioxidant and immune activities of *Oreochromis niloticus* fingerlings

**DOI:** 10.1186/s12917-024-04058-z

**Published:** 2024-05-21

**Authors:** El-Sayed Hemdan Eissa, Marwa S. Khattab, Samia Elbahnaswy, Gehad E. Elshopakey, Muna Omer Alamoudi, Rabab Mohamed Aljàrari, Mohammad B. Munir, Zulhisyam A. Kari, Mohammed A.E. Naiel

**Affiliations:** 1 Fish Research Centre, Faculty of Agricultural Environmental Sciences, Arish University, El-Arish, 45511 Egypt; 2 Department of Pathology, Faculty of Veterinary Medicine, Cairo University, Giza, 12211 Egypt; 3 Department of Aquatic Animal Medicine, Faculty of Veterinary Medicine, Mansoura University, Mansoura, 35516 Egypt; 4 Department of Clinical Pathology, Faculty of Veterinary Medicine, Mansoura University, Mansoura, 35516 Egypt; 5 Biology Department, Faculty of Science, University of Ha’il, P.O. Box 2440, Ha’il, 2440 Saudi Arabia; 6 Department of Biology, College of Science, University of Jeddah, Jeddah, 21959 Saudi Arabia; 7 Faculty of Agriculture, Universiti Islam Sultan Sharif Ali, Sinaut Campus, Tutong, TB1741 Negara Brunei Darussalam; 8 Department of Agricultural Sciences, Faculty of Agro-Based Industry, Universiti Malaysia Kelantan, Jeli Campus, Jeli, 17600 Malaysia; 9 Animal Production Department, Faculty of Agriculture, Zagazig University, Zagazig, 44511 Egypt

**Keywords:** Antioxidant, Growth, Immunity, Nile tilapia, Nanocurcumin *Spirulina platensis*

## Abstract

**Context:**

Recently, prioritize has been given to using natural phytogenic or nano compounds as growth promoters and immunostimulants in fish diets as an alternative to antibiotics.

**Aims:**

The main propose of this trial was to determine the impact of supplementing diets with spirulina or curcumin nanoparticles on the performance and health indicators of Nile tilapia fingerlings.

**Methods:**

In a 56-day feeding trial, 180 tilapia fingerlings were assigned into three main groups, as follows: 1st, control group, 2nd, *Spirulina platensis* (SP; 5 g kg^-1^ diet) and 3rd, curcumin nanoparticles (CUR-NPs; 30 mg kg^-1^ diet).

**Key results:**

Incorporating tilapia diets with SP or CUR-NPs significantly improved performance, body chemical analysis, blood biochemical and hematological indices, digestive enzyme activities, and antioxidant and immunostimulant features compared to the control.

**Conclusion:**

Fortified tilapia diets with CUR-NPs or SP efficiently boost the productivity and health of Nile tilapia fingerlings.

**Implications:**

The research introduces new practical solutions for applying safe feed additives as alternatives to antibiotics in tilapia farming.

## Introduction

Nile tilapia (*Oreochromis niloticus*) is the third-most cultivated fish species in aquaculture, behind grass carp and silver carp [1]. In Africa, both in its native habitat and farmed areas, it is typically the most valuable fish species captured from both inland fisheries and aquaculture mass production [2–4]. Especially in developing countries, *Oreochromis niloticus* is ideal for intensive or extensive aquaculture systems, due to its rapid growth, tolerance of a wide range of ecological conditions, resistance to diseases and stress, ability to reproduce under captive conditions, short generation time, low-trophic feeding, and acceptance of artificial feeds [5, 6]. Intensive tilapia farming encourages disease outbreaks [7]. In particular, environmental factors, fish health, and pathogen variables impact the immune system, enabling pathogens to spread diseases [8].

Recently, there has been a growing interest in employing natural phytogenic compounds as growth promoters and antimicrobials in aquatic animal diets [9–11] in order to minimize the application of antibiotics that lead to resistant strains of bacteria [12, 13], as well as their accumulation in edible tissues [14, 15]. Synthetic or natural immunostimulants (such as, glucans, probiotics, trace elements, cytokines, hormones, and products derived from animals, algae, and herbs) can effectively stimulate fish growth, immunological response, and resistance to environmental stressors [16]. However, the administration of hormones, vitamins, and chemical products is frequently not recommended because they could cause adverse reactions in fish health and generate potentially hazardous residues for consumers [17–19]. Set against this, plant-based constituents might be successful vaccinations instead of common drugs and antibiotics since they provide physiologically active secondary metabolites that are easily accessible, cheap, and nontoxic [20, 21].

Curcumin (CUR), known as diferuloylmethane, is a polyphenolic and hydrophobic chemical substance derived from the plant *Curcuma longa* [22]. Nutritionally, the CUR dried powder contains 60–70% carbohydrates, 3–6% fiber, 3–6% terpenes and terpenoids, 6–10% fat, and 6–8% protein [23, 24]. Curcumin continues to attract a lot of interest in aquaculture owing to the vast range of pharmacological characteristics it shows, such as its anti-lipid-accumulation, anticancer, antioxidant, anti-inflammatory, and antiviral features [12, 25, 26]. A recent investigation has revealed that incorporating curcumin into the Nile tilapia diet may promote growth and boost resistance against bacterial diseases [2, 27]. Moreover, dietary CUR intake may improve rainbow trout (*Oncorhynchus mykiss*) immunological function, performance, and antioxidant capacity [28]. Moreover, CUR may diminish the liver damage and apoptosis that chlorpyrifos causes in largemouth bass (*Micropterus salmoides*) [29]. Despite its benefits, CUR does have some drawbacks, such as the fact that it is weakly absorbed by the body because of its limited aqueous solubility, also known as hydrophobic, and unbalanced molecular assembly and that different species and sexes have different levels of bioavailability or utilization [30]. To boost its bioavailability and solubility, lipophilic curcumin can be transformed into nanoparticles, as has been amply demonstrated [31]. In comparison to the standard form of the molecule, nano curcumin demonstrated improved solubility and absorption rates [32, 33]. According to a prior study, consuming feed supplemented with 0.2% nano curcumin significantly enhanced the mucosal immune system, antioxidant capacities, and glucose homeostasis in the largemouth seabass [34]. Meanwhile, supplementing Nile tilapia diets with curcumin nanoparticles enhanced antioxidant capability, humoral immunity, hepatic and intestinal histology, as well as other blood biochemical measurements [2]. Nonetheless, further research is necessary to determine the effect of nano-curcumin on performance and general health status of Nile tilapia fingerlings.

In freshwater and marine environments, microalgae are referred to as filamentous organisms [35]. They have been utilized for nutrition and animal feeding for a very long time [36, 37]. Spirulina, *Arthrospira platensis*, a filamentous cyanobacterium, is known as a blue-green microalga and has a highly nutritious profile with 60–70% vitamins and proteins [38]. Moreover, it was found to be a trustworthy supplier of protein for farmed fish [39]. In addition to basic protein, amino acids, B vitamins (mainly riboflavin), polyunsaturated fatty acids (PUFA), chlorophyll, necessary minerals (particularly iron), carotenoids, minerals, and other nutritional components, spirulina also includes two pigments called phycocyanin and allophycocyanin [40, 41]. Spirulina has been shown to perform effectively with tilapia at up to 40% of their protein requirements, or around 12% of the overall diet, although higher amounts seem to influence palatability [42]. Thus, spirulina is usually utilized at lower rates, ranging from 0.5 to 3% of the entire diet, to improve coloring, fertility, and immune system function [43]. Several recent investigations revealed the efficacy of Spirulina supplementation in promoting Nile tilapia growth performance, immunological reactivity, and resistance to infections [44–47]. Moreover, *S. platensis* has been displayed to boost the immunity system and illness struggle of the African catfish (*Clarias gariepinus*) [48], due to the pigments’ presence, which has antioxidative benefits and the ability to eliminate free radicals.

Therefore, the current trial aimed to determine the effect of incorporating *S. platensis* or curcumin nanoparticles on the performance, redox status, organ histology, blood hematological and biochemical indices, digestive enzyme activities, and immunological responses of Nile tilapia fingerlings.

## Materials and methods

### Preparation of curcumin nanoparticles (CUR-NPs)

**Fig. 1:**
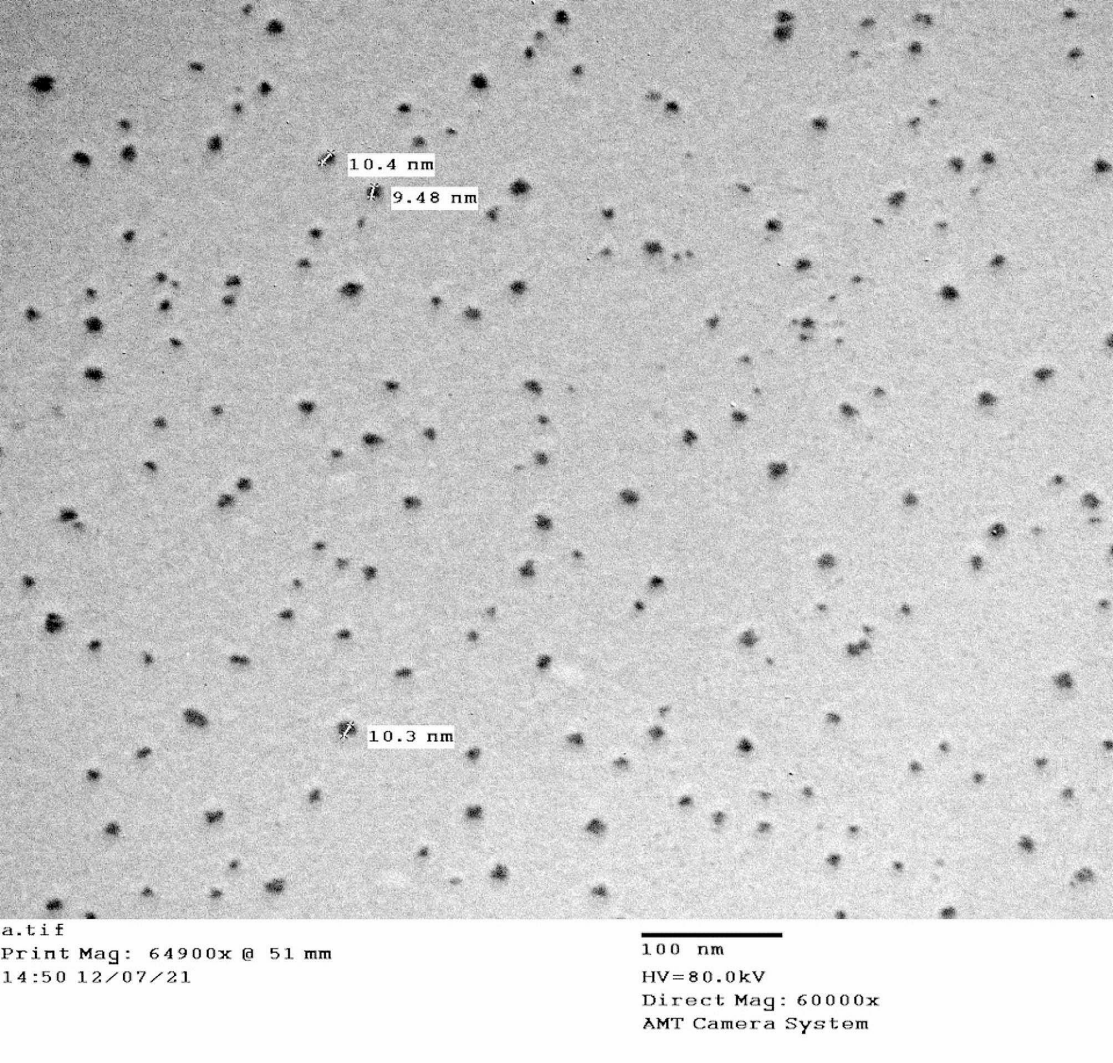
A TEM image of curcumin nanoparticles reveals spherical particles of various sizes and few aggregations, Scale bar = 82.7 ± 11.1 nm

With a few modest modifications, a method was used to create CUR-NPs using a syringe pump that included antisolvent [49]. Dichloromethane was the organic solvent used [50]. The first curcumin solution (240 mg) was dissolved into 40 mL of dichloromethane and put into a syringe (20 mL) before being injected (10 mL per min) at a 1:12 ratio into the antisolvent/deionized water while being agitated magnetically (one kg) for two hours at 1200 rpm. Then, the NPs were created utilizing a dry procedure by vacuum and filtered. The generated CUR-NPs powder was redissolved in deionized water at the required examining dosage (30 mg/mL CUR-NPs). The CUR-NPs dimension was calculated by employing a Zeta sizer (Malvern Instruments, Zeta sizer nano series Nano-s, UK). The average particle diameter was 82.7 ± 11.1 (Fig. 1).

### Dietary feeding preparation

Based on the nutritional demands of fish, three isocaloric (3000 Kcal DE/kg) and isonitrogenous (32%) diets have been created to suit the dietary needs of Nile tilapia [51]. In this study, three different types of diets were employed as dietary groups: a control diet with no feed additives, a diet containing CUR-NPs (30 mg kg^-1^) [2], and a diet containing SP (*S. platensis*; 5 g kg^-1^) [52]. The diet ingredients of the control and supplemented groups as well as their proximate chemical composition are shown in (Table 1). The formulated diets were thoroughly mixed with distilled water (400 g kg^-1^ diet) and pellets were prepared using a moist pellet machine without drying to obtain sinking pellets that could float in water. The pellets were air-dried to a moisture content of about 10%, and then remained in plastic bags and preserved in the refrigerator (-20 °C) until use.

### Rearing fish

The experimental fish were bought from a private fish hatchery located in Kafr El-Shiekh Governorate, Egypt, and then delivered via plastic bags to the fish diseases and management laboratory at Mansoura University. During two weeks, 180 mono-sex Nile tilapia fingerlings (10 ± 0.5 g each) were kept so they could become acclimated to the laboratory environment. During the acclimation period, fish were given the control diet 3 times/day, with 30% crude protein (CP) until apparent satiation. Following acclimatization, the fish were divided into three equal groups (20 fish in each 100-L tank) in three replicates. The first group was provided the standard diet and acted as the control group, while the other two groups received diets supplemented with 30 mg kg^-1^ CUR-NPs or 5 g kg^-1^*Spirulina platensis* (SP). The feeding experiment was prolonged for 56 days.

Air pumps and air stones were employed to provide oxygen to the tank water. Throughout the feeding trial period, the fish were given feed three times each day at 4-hour intervals (at 9:00, 13:00 and 17:00 h). Each tank’s trash was removed daily, with half of it being disposed of in freshwater.

Throughout the study, fluorescent light cylinders were employed to keep a twelve-hour cycle of dark and light going. Unionized ammonia levels were checked twice a day through an automated probe (Hanna HI-9147). The water temperature, pH, and dissolved oxygen levels were estimated daily using particular portable equipment to be kept within acceptable range for tilapia production as ascribed by Boydand Tucker [53], as follows: 27.0–29.2 °C, 6.2–6.9 mg/L, and 7.95–8.37 mg/L, respectively.

**Table 1:** Composition and proximate analysis of control and supplemented diets (% dry matter) of Nile Tilapia with curcumin nanoparticles (CUR-NPs) and *Spirulina platensis* (SP) for 56 days

Ingredients	Control	CUR-NPs	SP
Fish meal (CP 72%)	110	110	110
Soybean meal (CP 48%)	360	360	360
Rice bran	200	200	200
Wheat bran	200	200	200
Yellow corn	60	59.97	55
Nano curcumin	----	0.03	----
Spirulina	-----	----	5
Fish Oil	15	15	15
Soybean Oil	15	15	15
Molasses	20	20	20
Dicalcium phosphate	10	10	10
Vitamins ^1^ & Minerals ^2^ premix	10	10	10
Total	1000	1000	1000
Proximate analysis (% dry matter basis)			
Dry matter	91.5	91.48	91.42
Crude Protein (CP)	30.29	30.28	30.32
Crude Fat	8.17	8.16	8.17
Crude Fiber	6.62	6.61	6.61
Carbon-free nitrogen extract (NFE)^2^	47.7	47.71	47.7
Ash	7.23	7.21	7.22
Gross energy (GE, MJ/Kg diet)^3^	18.57	18.57	18.58

^1^Vitamin premix (per kg of premix): thiamine, 2.5 g; riboflavin, 2.5 g; pyridoxine, 2.0 g; inositol, 100.0 g; biotin, 0.3 g; pantothenic acid, 100.0 g; folic acid, 0.75 g; para-aminobenzoic acid, 2.5 g; choline, 200.0 g; nicotinic acid, 10.0 g; cyanocobalamin, 0.005 g; a-tocopherol acetate, 20.1 g; menadione, 2.0 g; retinol palmitate, 100,000 IU; cholecalciferol, 500,000 IU

^2^ Mineral premix (g/kg of premix): CaHPO_4_. 2H_2_O,727.2; MgCO_4_.7H_2_O, 127.5; KCl 50.0; NaCl, 60.0; FeC_6_H_5_O_7_.3H_2_O, 25.0; ZnCO_3_, 5.5; MnCl_2_. 4H_2_O, 2.5; Cu (OAc)_2_. H_2_O, 0.785; CoCl_3_.6H_2_O, 0.477; CaIO_3_. 6H_2_O, 0.295; CrCl_3_. 6H_2_O, 0.128; AlCl_3_.6H_2_O, 0.54; Na_2_SeO_3_, 0.03. CP; Crude protein

^3^Gross energy (GE) was calculated from NRC (2011) as 23.6 kJ/g, 39.4 kJ/g, and 17.2 kJ/g for protein, lipid, and carbohydrates, respectively

### Samples collecting and preparing the homogenate tissues

The feeding study included sampling fish 24 h after they had last eaten. Five fish from every group were anesthetized with 60 mg/L of clove oil. The caudal blood vessels of each fish were used to collect blood samples [54]. A single blood sample was taken using disposable syringes having a dipotassium EDTA solution for hematological indices assay. Centrifugation at 3000 g min. at 4 °C for 15 min was used to separate the serum from the other blood sample. The serum was then maintained at -20 °C for blood biochemistry, immunological, and antioxidant indices examination. Non-heparinized disposable syringes were applied to pick up this sample of blood. The fish belly was dissected to get the organs (gills, intestine, stomach, kidney, and liver) following Pirarat et al. [55] procedure.

### Growth and feed variables, and survival percentage

After the trial, fish were taken out of each tank, numbered, and weighed. The formulas described below were utilized to construct the factors affecting fish growth and feed efficiency indices [5, 56]:

$$\begin{gathered} {\text{Weight}}\,{\text{gain}}\,{\text{percentage}}\,\left( {{\text{WG}}} \right)\, \hfill \\ =\,100\, \times \,\left( {\left( {{{\text{W}}_2}\,--\,{{\text{W}}_1}} \right)\,/\,{{\text{W}}_1}} \right) \hfill \\ \end{gathered}$$

$$\begin{gathered} {\text{Specific}}\,{\text{growth}}\,{\text{rate}}\,\left( {{\text{SGR;}}\,{\text{\% g}}\,/\,{\text{day}}} \right) \hfill \\ \,=\,100{\text{ }}\left[ {{\text{Ln}}\,{{\text{W}}_2}\left( {\text{g}} \right)\,--\,{\text{Ln}}\,{{\text{W}}_1}\left( {\text{g}} \right)} \right]\,/\,{\text{T}} \hfill \\ \end{gathered}$$

where W1 denotes the starting weight, W2 is the ending weight, and T is the trial time (day).

$$\begin{gathered} {\text{FCR}}\,\left( {{\text{Feed}}\,{\text{conversion}}\,{\text{ratio}}} \right) \hfill \\ {\text{=}}\,{\text{feed}}\,{\text{consumption}}\,{\text{in}}\,{\text{g}}\,{\text{/}}\,{\text{WG}}\,{\text{in}}\,{\text{g}} \hfill \\ \end{gathered}$$

Throughout the feeding trial period, the unconsumed pellet was gently gathered from all tank bottoms after 30 min of handled feed. The exact feed intake amount was calculated by subtracting the dry mass of uneaten pellets from the calculated provided feed (FI). While, the daily diet allowance for each fish group was changed relying on the control group feed intake from the day before.

$$\eqalign{& {\rm{Fish}}\,{\rm{survival}}\,\left( {\rm{\% }} \right)\, = \cr & \left( {{\rm{fish}}\,{\rm{number}}\,{\rm{at}}\,{\rm{end}}\,{\rm{of}}\,{\rm{trial}}} \right. \cr & \left. {{\rm{/fish}}\,{\rm{number}}\,{\rm{at}}\,{\rm{the}}\,{\rm{start}}\,{\rm{of}}\,{\rm{trial}}} \right) \cr & \times 100 \cr}$$

### Analyzing the composition of the whole body

Fish samples (*n* = 8/group) were taken during the harvesting period and stocking for the preliminary and conclusive proximate carcass investigations, respectively. The approved methods were used to perform the proximate analysis according to previous investigations [57]. The crude protein (CP) was quantified by Kjeldahl nitrogen, and crude fat was determined by weighing the sample after being extracted with petroleum ether (40–60 °C). To determine the quantity of ash, dry samples were burnt at 550 °C for 4 h in a muffle furnace.

### Hematological investigations

Leukocytes and erythrocytes were physically numbered after becoming diluted in Natt- Herrick’s solution [58]. These modified techniques were also used to assess the indicators of red blood cells, packed cell volume, and hemoglobin [59, 60]. Blood films were additionally stained with Wright’s Giemsa to estimate the variation in leukocytic count [58].

### Serum biochemical analysis

Fish blood glucose levels were measured using a modified technique stated by Wedemeyer and Yasutake [61]. Total protein (TP; MBS9917835), albumin (MBS038444), alanine aminotransferase (ALT; MBS019237), triglycerides (MBS9719080), alkaline phosphatase (ALP; MBS033204), were assessed spectrophotometrically using ELISA kits (Lambda EZ201; Perkin Elmer) acquired form My-Bio-Source Company (California, USA) while assessing the activity of cholesterol levels (EK12283), and aspartate aminotransferase (AST; EK12276) were determined via specific kits (Biotrend Co. Maryland, USA) following the pamphlet guidelines. Moreover, the amount of uric acid and creatinine were measured as previously mentioned [61–63], respectively.

### Immune assays

Evaluation of serum lysozyme action was done using *Micrococcus lysodeikticus* lysis [64], with little modifications (Sigma Co., USA). Then the OD (optical density) was defined at 540 nm once per minute for 5 min. Numerous dilutions of lyophilized chicken egg-white lysozyme were exploited to build up a standard curve to identify the serum lysozyme level (Sigma Co., USA). The immunoglobulin M (IgM) levels in serum were also tested following the manufacturer guidelines purchased from Cusabio Co., Texas, USA, Catalog No. CSB-E12045Fh).

### Activity of intestinal digestive enzymes

Using the methods used in prior studies [65–67], the intestinal homogenate supernatant’s amylase, lipase, and protease enzyme activity were each measured.

### Status of antioxidants and oxidative stress

The concentrations of four antioxidants from the serum of each group were calculated, including glutathione peroxidase (GPx) enzyme, malondialdehyde (MDA) enzyme, catalase (CAT), and superoxide dismutase (SOD) using the approaches previously reported [68–72].

### Histological examination

Gills, stomach, kidney, intestine, and liver specimens were all well-kept in 10% neutral buffered formalin. The tissues were subsequently subjected to ethanol and xylene processing in escalating concentrations, submerged in paraffin, and sliced into pieces measuring 4 μm thick using a rotary microtome [73]. The tissue slices were stained with hematoxylin and eosin [74], and the tissue was examined and captured on camera with a light microscope (Olympus, Tokyo, Japan).

### Analytical statistics

Using the SPSS® program, version 26.0, statistical analysis was performed (IBM Corporation, SPSS Statistics, USA). The trial’s results were displayed as a standard error of the mean (means ± SE). Levene’s test was used to initially assess all data for variance, normality, and homogeneity. The mean values for each experimental group were then compared using a one-way ANOVA and post-hoc Duncan’s multiple range testing. The findings on digestive, immunological, and antioxidant enzymes were examined using one-way ANOVA with post-hoc Tukey’s multiple range testing (*P < 0.05, **P < 0.01; ***P < 0.001). The GraphPad Prism software (version 8.0) was utilized to graphically display results.

## Results

### Impact of CUR-NPs and SP supplements on growth performance, survival percentage, and feed utilization indices

The growth characteristics, feed intake, and survival % of fish diets supplemented by SP or CUR-NPs were summarized in Table 2. Fish assigned diets containing SP or CUR-NPs showed statistically significant increases (*P* ≤ 0.05) in specific growth rate (SGR), weight gain (WG), weight gain percent (WG%), total consumed feed, final body weight (FBW) and total fish biomass compared to the control group. Whilst, both fish groups provided supplemented diets (SP or CUR-NPs) had significantly lower FCR (*P* ≤ 0.05) than the control group. On the other hand, the survival rates in all examined groups were not statistically different from one another.

**Table 2:** Growth performance and feed utilization of Nile tilapia-fed diets supplemented with curcumin nanoparticles (CUR-NPs) and *Spirulina platensis* (SP) for 56 days

Parameters^1^	Control	CUR-NPs	SP	*P* value
Initial body weight (g)	10.00 ± 0.06	9.97 ± 0.03	10.00 ± 0.06	0.089
Final body weight (FBW, g)	24.77 ± 0.25^b^	35.85 ± 1.43^a^	37.43 ± 1.63^a^	0.021
Weight Gain (g)	14.77 ± 0.28^b^	25.88 ± 1.46^a^	27.43 ± 1.69^a^	0.014
Weight Gain %	147.67 ± 1.38^b^	259.67 ± 1.32^a^	274.57 ± 1.48^a^	0.007
SGR (% day^− 1^)	1.62 ± 0.03^b^	2.28 ± 0.08^a^	2.45 ± 0.05^a^	0.024
Feed intake (g feed/fish)	30.59 ± 0.13^b^	33.63 ± 0.51^a^	34.13 ± 0.37^a^	0.001
FCR (g/g)	2.07 ± 0.03^a^	1.31 ± 0.05^b^	1.25 ± 0.06^b^	0.001
Fish biomass (g fish)	495.33 ± 4.91^b^	705.87 ± 3.55^a^	748.67 ± 2.62^a^	0.002
Survival rate (SR,%)	100 ± 0.33	100 ± 0.67	100 ± 0.31	0.061

^1^Values are expressed as a standard error of the mean (means ± SE). Data in the same row assigned with the different superscripts are significantly different (*p* < 0.05) using ANOVA Post Hoc (Duncan test). FCR, feed efficiency ratio; SGR, specific growth rate; SR, survival rate

### A body chmical composition investigation’s response to the addition of CUR-NPs or SP

Table 3 exhibits the body chemical analysis of *O. niloticus* after being administered SP or CUR-NPs. Fish given a diet enriched with CUR-NPs exhibited significantly greater amounts of DM (dry matter) (*P* ≤ 0.05) than those provided with the control diet. In the same tend, supplemented diets with SP improved ash and protein contents significantly (*P* ≤ 0.05) compared to other treatment groups. In contrast, the experimental groups demonstrated no significant differences in terms of crude fat content.

**Table 3:** Carcass proximate composition of Nile tilapia fed with curcumin nanoparticles (CUR-NPs) or *Spirulina platensis* (SP) for 56 days

Compositions (g kg^− 1^)*	Control	CUR-NPs	SP	*P* value
DM (%)	25.25 ± 0.05^c^	27.33 ± 0.10^a^	27.01 ± 0.05^b^	0.001
Protein (%)	57.13 ± 0.55^c^	59.63 ± 0.06^b^	60.94 ± 0.08^a^	0.014
Crude fat (%)	25.49 ± 0.60	24.37 ± 0.07	24.61 ± 0.05	0.155
Ash (%)	14.44 ± 0.05^c^	15.21 ± 0.04^b^	15.40 ± 0.02^a^	0.033

*Values are expressed as means ± SE. Data in the same row assigned with the different superscripts are significantly different (*p* < 0.05) using ANOVA Post Hoc (Duncan test), DM; dry matter

### Hematological analysis of CUR-NPs and SP supplementation

Relative to the control fish group, fish given diets enriched with SP and CUR-NPs exhibited a considerable improvement (*P* ≤ 0.05) in RBC count, Hb levels, and Ht%. MCH, although they failed to show any significant group differences. Furthermore, total leukocyte (WBC) counts were significantly higher (*P* ≤ 0.05) in the CUR-NPs-treated fish relative to the control and SP treatments. Moreover, monocytes and neutrophils in the SP and CUR-NPs groups were considerably increased (*P* ≤ 0.001), but lymphocytes in the control fish were significantly reduced (*P* ≤ 0.001), as shown in (Table 4).

**Table 4:** Hematological parameters of Nile tilapia-fed diets supplemented with curcumin nanoparticles (CUR-NPs) or *Spirulina platensis* (SP) for 56 days

Items*	Control	CUR-NPs	SP	*P* value
RBCs (1 × 10^6^ uL)	3.12 ± 0.05^b^	3.73 ± 0.02^a^	3.64 ± 0.08^a^	0.005
Hb (mg/dL)	7.37 ± 0.42^b^	9.37 ± 0.19^a^	8.83 ± 0.26^a^	0.001
MCH (%)	23.60 ± 1.42	25.14 ± 0.61	24.52 ± 1.07	0.245
Ht (%)	39.00 ± 2.80^b^	50.93 ± 0.23^a^	48.50 ± 2.72^a^	0.002
WBCs (x10^3^ uL)	18.13 ± 0.94^b^	22.07 ± 1.12^a^	19.40 ± 0.53^ab^	˂0.001
Lymphocytes (%)	65.37 ± 0.74^a^	51.23 ± 1.96^b^	54.53 ± 1.10^b^	˂0.001
Neutrophils (%)	22.50 ± 0.61^b^	30.47 ± 1.60^a^	28.20 ± 1.10^a^	˂0.001
Monocytes (%)	11.40 ± 0.10^b^	19.20 ± 0.51^a^	18.30 ± 1.10^a^	˂0.001

*Values are expressed as means ± SE. Data in the same row assigned with the different superscripts are significantly different (*p* < 0.05) using ANOVA Post Hoc (Duncan test). RBCs; Red blood cells, Hb; hemoglobin, MCV; mean corpuscular volume, Ht; hematocrit and WBCs, white blood cells

### Impact of serum biochemical variables after treatment with SP and CUR-NPs

Table 5 displays the effects of SP or CUR-NPs-supplemented diets on the blood biochemistry parameters of Nile tilapia fingerlings. In both supplemented groups (SP or CUR-NPs addition), glucose and cholesterol levels significantly decreased (*P* ≤ 0.05) compared to the control fish. Moreover, triglycerides, total protein, albumin and globulin were considerably higher in the SP treatment relative to the control and CUR-NPs treatments (*P* ≤ 0.001). Moreover, both SP and CUR-NPs groups had significantly greater concentrations of total protein, globulin, and albumin, compared with the control ones (*P* ≤ 0.05). In contrast to the control group, both activities of ALP and AST were also reduced in both SP and CUR-NPs treatments. While, no significant alterations (*P* ≤ 0.05) were found regarding ALT, creatinine level, and uric acid in the Nile tilapia serum for all experimental groups.

**Table 5:** Serum-biochemical parameters of Nile tilapia-fed diets supplemented with curcumin nanoparticles (CUR-NPs) or *Spirulina platensis* (SP) for 56 days

Items*	Control	CUR-NPs	SP	*P* value
Glucose (mg/dL)	113.80 ± 5.08^a^	89.37 ± 0.90^b^	93.47 ± 2.43^b^	0.011
Total cholesterol (mg/dL)	192.67 ± 5.24^a^	180.17 ± 2.37^b^	173.43 ± 0.29^b^	0.007
Triglycerides (mg/dL)	152.77 ± 2.39^b^	147.53 ± 3.67^b^	170.43 ± 2.43^a^	˂0.001
Total protein (g/dL)	4.09 ± 0.04^c^	5.22 ± 0.04^b^	5.77 ± 0.03^a^	˂0.001
Albumin (g/dL)	2.36 ± 0.01^c^	2.49 ± 0.02^b^	2.77 ± 0.02^a^	˂0.001
Globulin (g/dL)	1.73 ± 0.03^c^	2.73 ± 0.02^b^	3.00 ± 0.01^a^	˂0.001
AST (IU/L)	12.40 ± 0.10^a^	12.13 ± 0.09^ab^	11.87 ± 0.09^b^	0.014
ALT (IU/L)	41.87 ± 0.91	42.20 ± 0.90	39.43 ± 0.84	0.876
ALP (IU/L)	31.20 ± 0.25^a^	30.33 ± 0.24^b^	28.57 ± 0.20^c^	0.041
Creatinine (mg/dL)	1.75 ± 0.10	1.81 ± 0.03	1.81 ± 0.02	0.097
Uric acid (mg/dL)	0.53 ± 0.01	0.53 ± 0.01	0.52 ± 0.02	0.371

*Values are expressed as means ± SE. Data in the same row assigned with the different superscripts are significantly different (*p* < 0.05) using ANOVA Post Hoc (Duncan test). ALT; alanine transaminase, ALP; alkaline phosphatase and AST; aspartate aminotransferase

### Immune parameters are affected by the addition of CUR-NPs and SP

**Fig. 2:**
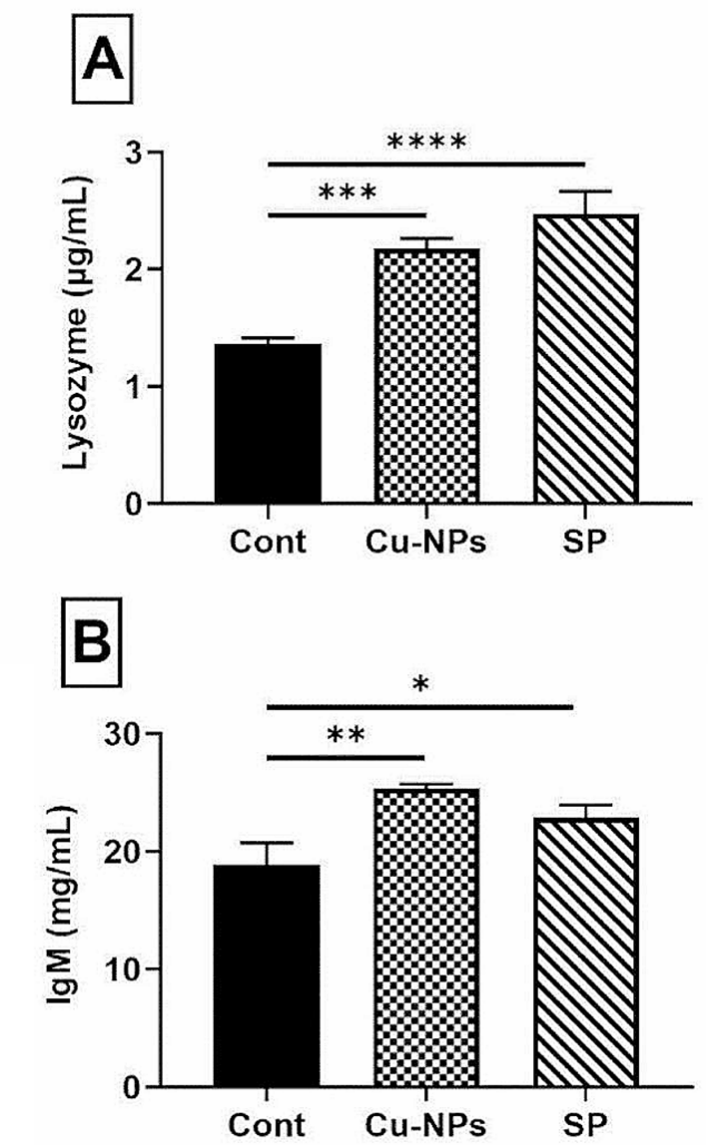
Impacts of CUR-NPs (30 mg kg^-1^) or SP (5 g kg^-1^) dietary supplementation on Nile tilapia immune activities regarding lysozyme activity (**A**), and Ig M levels (**B**). Data were presented as mean ± SE (*n* = 5). The one-way ANOVA was used using Tukey’s post hoc test to determine the significant variance. *, **, ***, **** were significant differences at *P* ≤ 0.05, *P* ≤ 0.01 and *P* ≤ 0.001, *P* ≤ 0.0001, respectively. Three groups were named as; Cont. control group, CUR-NPs and SP, dietary supplementation with curcumin nano-particles or *Spirulina platensis*, respectively

As shown in Fig. 2, the addition of SP or CUR-NPs remarkably increased fish immune responsive system, as shown by a considerable increase in the activity of Ig M (*P* ≤ 0.01) and lysozyme activity (*P* ≤ 0.001) in contrast to those of the control ones.

### Impacts of intestinal digestive enzyme activities upon CUR-NPs and SP- supplements

**Fig. 3:**
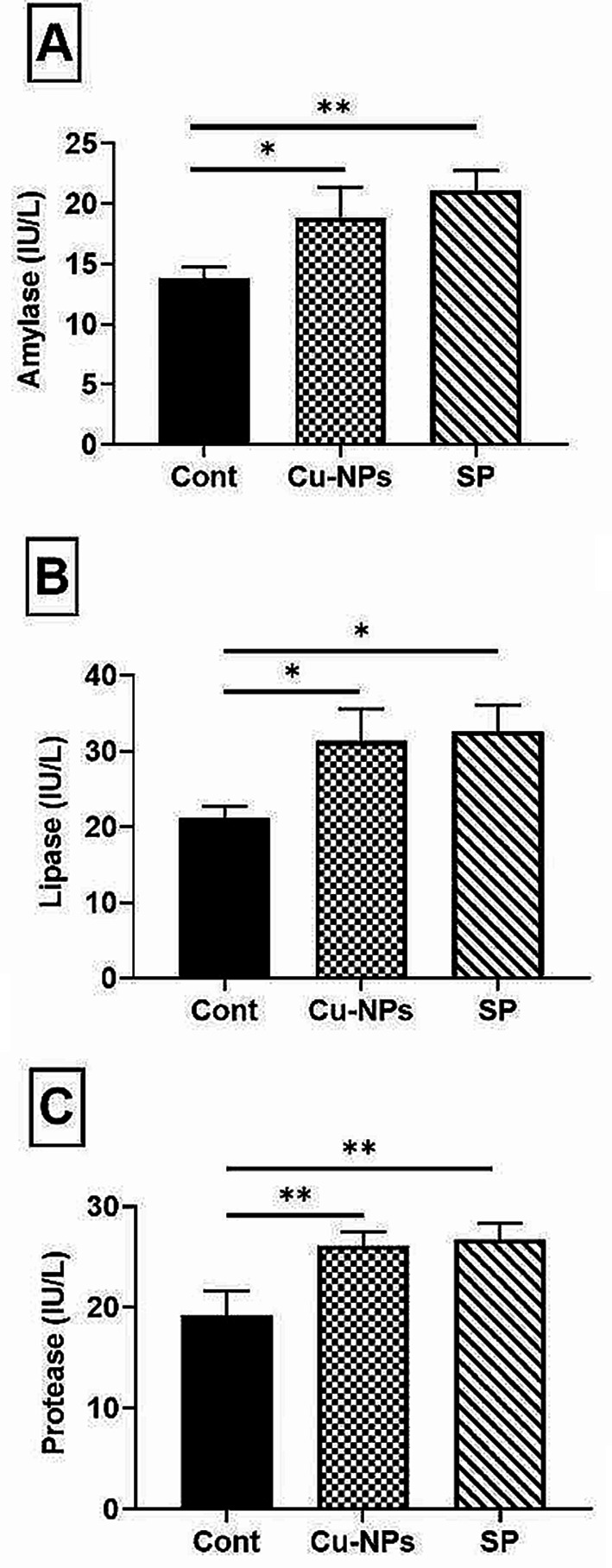
Impacts of CUR-NPs (30 mg kg^-1^) or SP (5 g kg^-1^) dietary supplementation on Nile tilapia intestinal digestive enzymes activities regarding amylase (**A**), lipase (**B**), and protease (**C**). Data were presented as mean ± SE (*n* = 5). The one-way ANOVA was used using Tukey’s post hoc test to determine the significant variance. *, ** were significant differences at *P* ≤ 0.05, and *P* ≤ 0.01, respectively. Three groups were named as; Cont. control group, CUR-NPs and SP, dietary supplementation with curcumin nano-particles or *Spirulina platensis*, respectively

Fish given a meal containing SP or CUR-NPs had significantly stimulated (*P* ≤ 0.05 or 0.01) intestinal amylase, protease, and lipase activities when compared to the control fish group (Fig. 3), demonstrating that CUR-NPs and SP supplements are efficient in promoting fish digestion process and feed efficiency.

### Redox state after supplementing with CUR-NPs or SP

**Fig. 4:**
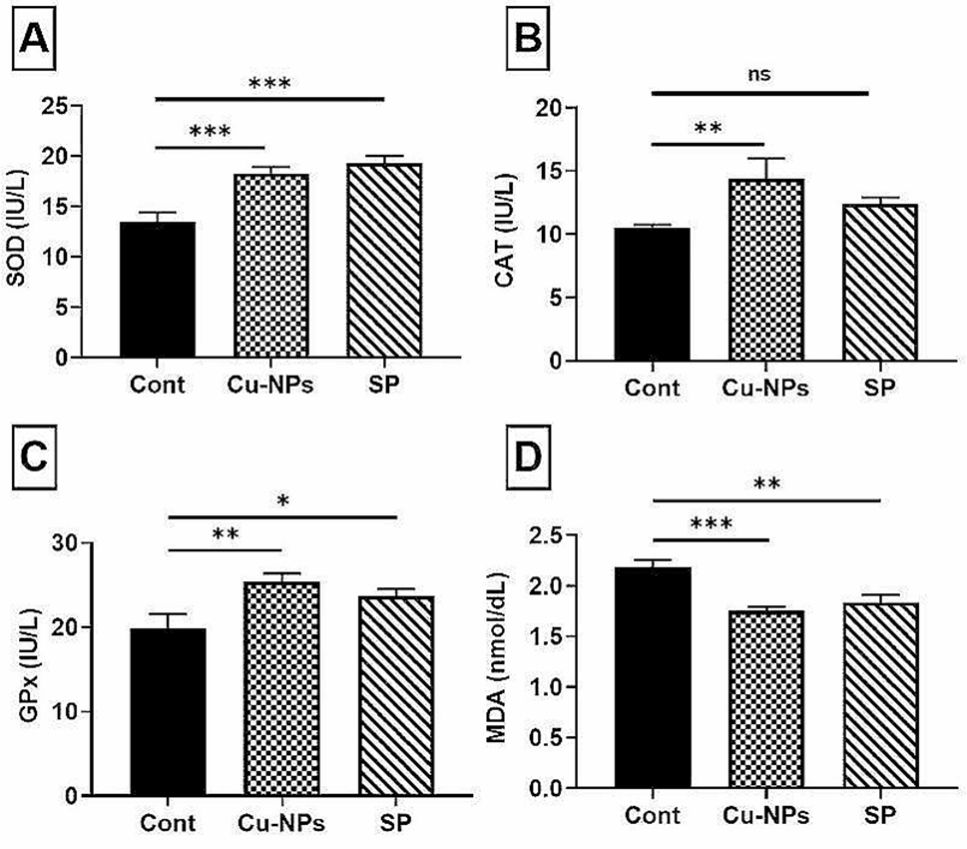
Impacts of CUR-NPs (30 mg kg^-1^) or SP (5 g kg^-1^) dietary supplementation on Nile tilapia antioxidant activities regarding (**A**) SOD, (**B**) CAT, (**C**) GPX and (**D**) MDA. Data were presented as mean ± SE (*n* = 5). The one-way ANOVA was used using Tukey’s post hoc test to determine the significant variance. Ns, *, **, *** were significant differences at non-significant, *P* ≤ 0.05, *P* ≤ 0.01 and *P* ≤ 0.001, respectively. Three groups were named as; Cont. control group, CUR-NPs and SP, dietary supplementation with curcumin nano-particles or *Spirulina platensis*, respectively

Figure 4 illustrates the impact of dietary supplementation with CUR-NPs or SP-based diets on antioxidant status. CUR-NPs or SP supplementation resulted in significantly increased GPx (*P* ≤ 0.05 or 0.01) and SOD (*P* ≤ 0.01 or 0.001) activities compared to the control fish (Fig. 4A, C). Conversely, supplementing diets with CUR-NPs or SP significantly reduced MDA levels (*P* ≤ 0.01 or 0.001) compared to the control group (Fig. 4D). Nevertheless, fish fed with diets containing CUR-NPs showed significant improvement in serum CAT (*P* ≤ 0.01) compared to the SP treatment and control (Fig. 4B).

### Impact of CUR-NPs or SP Supplements on the tissue structure of fish gills, stomach, intestinal, hepatic and renal tissues

**Fig. 5:**
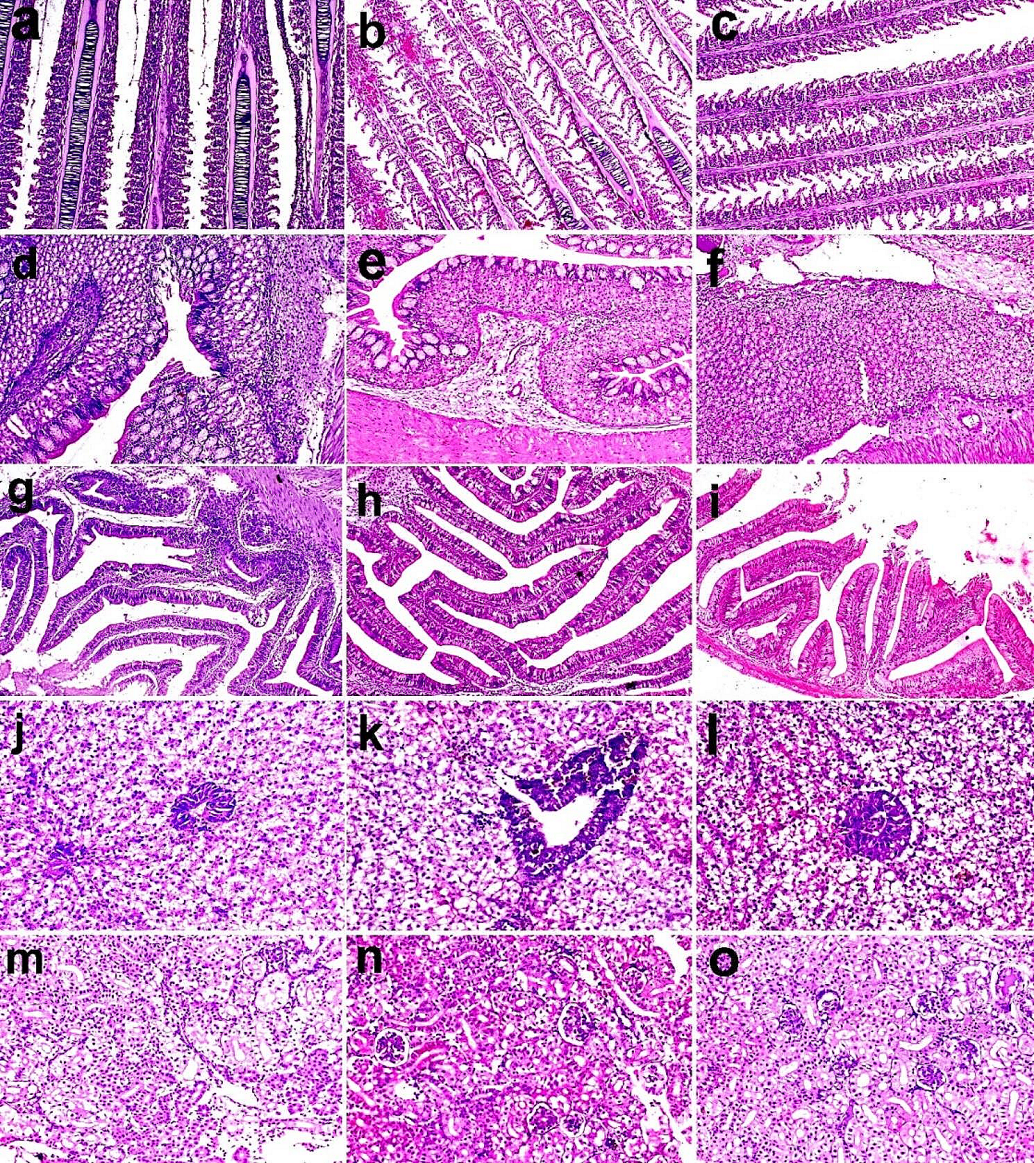
Representative photomicrograph of H&E-stained Sect. (50 μm) from gills (**a**, **b**, **c**), stomach (d, f,g), intestine (h, i,j), hepatopancreas (j, k,l) and spleen (m, n,o) of Nile tilapia fish fed un-supplemented diets (control), and CUR-NPs (30 mg kg^-1^) or SP (5 g kg^-1^) supplemented diets. (**a**) The fish group fed the control diet showed healthy gill structure with severe diffuse hyperplastic epithelium lining secondary gill lamellae. (**b**, **c**) the fish group fed diets supplemented with CUR-NPs or SP showed healthy gill structure with moderate hyperplasia of gill epithelium. (**d**) the fish group fed the control diet represented the normal structure of the gastric submucosa of the stomach with focal leukocyte infiltration and fibrosis. (**e**) the fish group fed CUR-NPs supplemented diets indicated healthy stomach tissue structure with moderate leukocyte infiltration in the gastric submucosa. While, (**f**) the fish group fed SP supplanted diets represented mild histopathological alteration in the stomach tissue structure with higher intensity of leukocyte infiltration in the gastric submucosa. (**g**) fed fish control diets show normal intestinal structure with low focal leukocyte infiltration in the submucosa and focal epithelial hyperplasia. (**h**, **i**) supplemented tilapia diets with CUR-NPs or SP show healthy and normal structure of intestinal mucosal, submucosal and epithelial cells. (**j**) The control group indicated moderate hepatocyte vacuolation in the liver structure, whereas, supplemented tilapia diets with CUR-NPs or SP (**k**, **l**) indicate lower hepatocyte vacuolation within hepatopancreatic tissues. (**m**, **n**, **o**) All evaluated renal tissues in all experimental groups revealed normal histological structure in the kidney

The secondary lamellae of the gill in the control group showed significant widespread hyperplasia of lamellar epithelium (Fig. 5a). In the CUR-NPs group, the hyperplasia of the lamellar epithelium was less severe compared to control (Fig. 5b). whereas, in the SP group, the gills had moderate hyperplasia of gill epithelium (Fig. 5c). Microscopy of the stomach in the control group revealed focal leukocyte infiltration and fibrosis in the gastric submucosa (Fig. 5d). In the CUR-NPs group, the leukocyte infiltration and fibrosis were less severe (Fig. 5e). While, in the SP group, the stomach microscopy revealed mild histopathological alteration with higher intensity of leukocyte infiltration in the gastric submucosa (Fig. 5-f). Microscopy of the intestine revealed focal leukocyte infiltration in the submucosa and focal epithelial hyperplasia in the control group (Fig. 5-g). These lesions were not observed in the CUR-NPs or SP groups (Fig. 5-h, i). Hepatopancreatic microscopy showed mild periportal leukocyte infiltration and moderate hepatocyte vacuolation in the control group (Fig. 5-j). In CUR-NPs or SP groups, the hepatocyte vacuolation was more severe compared to the control group (Fig. 5-k, l). Microscopy of the kidney revealed normal structure in all investigated groups (Fig. 5-m, n, o).

## Discussion

Applying natural nutritional supplements in aquafeed is an important technique for maintaining its sustainability [75, 76]. Numerous herbal remedies have been utilized in aquaculture and proven as growth promoters and immunostimulants [10, 19, 45, 76–78]. The current study results indicated that Nile tilapia given diets with CUR-NPs or SP for nearly two months displayed significant enhancement in growth and feed efficiency (FCR, and SGR) indices. Similar enhancements in growth variables were observed in Nile tilapia (*Oreochromis niloticus*) [1, 79, 80], Red tilapia [6], European seabass (*Dicentrarchus labrax*) [81], white-leg shrimp (*Litopenaeus vannamei*) [82] given diets containing CUR-NPs, as well as in gilthead seabream (*Sparus aurata*), rainbow trout (*Oncorhynchus mykiss*), common carp (*Cyprinus carpio*) and grass carp (*Ctenopharyngodon Idella*)fed CUR-based diets [83–86]. The growth-promoting impact of curcumin might be attributed to its ability to enhance digestive enzyme activity in the hepatopancreas and intestine, producing an improvement in nutrient absorption and utilization [87, 88]. Additionally, curcumin has a palatable flavor, which may increase feed intake and subsequently enhance feed efficiency [25].

The growth-promoting effects of *Spirulina, Arthrospira platensis* supplementation have been well-documented in Nile tilapia (*Oreochromis niloticus*) [47], Caspian brown trout (*Salmo trutta*) [89], and Oscar fish (*Astronotus ocellatus*) [78], leading to increased feed consumption and reduced nutrients retention. These effects are ascribed to the great concentration of bioactive composites in spirulina, including polysaccharides, γ-linolenic acid, polyunsaturated fatty acids, and pigments such as total carotenoids, chlorophyll, β-carotene, zeaxanthin, and phycocyanin [47, 89, 90]. Moreover, dietary fortification with SP has been shown to boost digestive enzyme activity in the intestinal of aquatic animals [90, 91]. Here, Nile tilapia feeding diets containing SP and Cu-NPs showed increased activity levels of lipase, amylase, and protease enzymes in their intestinal tract. Similar findings have been described in other reports investigating the effects of curcumin supplementation on the digestion-related enzyme activity of fingerlings of *Oreochromis mossambicus* [92], crucian carp (*Carassius carassius*) [88], and gilthead seabream larvae [93]. Recently, the addition of graded levels of CUR-NPs to fish feed for two months has been shown to enhance the growth indices of fish by enhancing digestive enzyme activity [2]. Additionally, SP supplementation has been found to enhance intestinal enzyme activity in rainbow trout (*Oncorhynchus mykiss*) [91, 94], and Oscar fish (*Astronotus ocellatus*) [78], leading to an increase in feed consumption and a reduction in nutrient retention.

In this study, dietary supplements of both CUR-NPs and SP were found to improve the fish body protein and ash contents after 56 days of treatment, with no significant variation in the lipid content. Similar results have been reported in recent studies investigating the long-term use of curcumin, which has been found to significantly increase the ash amount and protein levels in the muscles of Nile tilapia [87, 95]. The same trend was stated in Oscar fish feeding diets of SP powder [78]. Furthermore, SP supplementation has been shown to increase the ash amount in the dorsal muscles and the whole body while significantly decreasing the lipid concentration in juvenile gibel carp (*Carassius gibelio*) [96]. These findings are consistent with the data on growth performance distinguished in fish fed with CUR-NPs and SP diets.

The hematological parameters of fish provide valuable insight into the impact of herbal supplements on their nutritional and health status [28, 97]. The present study found that Nile tilapia supplied with SP and CUR-NPs diets showed an increase in RBC and WBC counts, such as Hb and Ht concentration, monocytes and neutrophils, compared to control fish, without any significant alters in MCH levels. These results are consistent with previous research on dietary curcumin and its nanoparticle analogs, which showed positive effects on hematological parameters in various fish species [2, 28, 83, 98].

The enhanced hematological markers indicate the positive effects of CUR-NPs and spirulina on Nile tilapia health, without causing anemia, by promoting hemosynthesis and erythropoiesis. This helpful association of curcumin with the metabolism and nutrients contained in Nile tilapia’s blood has been reported previously [83, 99, 100]. The increased levels of monocytes and neutrophils in diets containing CUR-NPs and CUR are liked to improve innate and adaptive immune response pathways in fish [2, 17, 101]. Curcumin may activate macrophages and neutrophils to produce reactive oxygen species (ROS), thereby increasing phagocytic activity and improving immunity [83, 102]. Similarly, *Spirulina platensis* has been shown to improve Hb, MCH, MCHC, and HCT in Oscar fish, attributed to the beneficial effects of phycocyanin on the bone marrow’s stem cells, promoting immune response and red blood cells in fish [78, 103].

The innate defense mechanism of fish includes lysozyme, a hydrolytic enzyme that triggers the phagocytes and the complement system, leading to the lysis of bacterial infections [104]. Serum immunoglobulins also play an important role in phagocytosis and decreasing the harmful microorganisms within the fish body [105]. In our study, Nile tilapia supplied with SP and CUR-NPs displayed a notable increase in the total Ig concentration and lysozyme levels. Previous research has shown that curcumin administration can augment IgM values in rainbow trout and promote serum immunity issues (lysozyme and IgM) in snakehead fish (*Channa argus*) [28, 106]. Rainbow trout, which had been given diets with curcumin had the highest lysozyme activity [107]. Curcumin nano micelles-enriched diets also increased lysozyme levels in White leg shrimp and Nile tilapia [2, 108]. Moreover, CUR-NPs given White leg shrimp showed a considerable increase in lysozyme mRNA transcription levels [82]. Similarly, *Spirulina platensis* has positive effects on the fish’s inherent immune response, such as lysozyme concentration [47, 78, 109]. Our results suggest that the activation of humoral immunity in Nile tilapia upon SP-diets and CUR-NPs administration may be attributed to curcumin’s ability to stimulate cytokine production and activate neutrophils and macrophages, thereby regulating the immune response of fish [102].

The association between fish immunity and antioxidant properties is well-established. Antioxidative enzymes like SOD, GPx, and CAT reduce oxidative stress by lowering reactive oxygen species (ROS) levels [93]. MDA, a marker for lipid peroxidation, reveals oxidative damage to lipids. Our study showed significant escalations in the activity of CAT, SOD, and GPx enzymes and a decline in MDA contents in fish fed with SP and CUR-NPs, indicating the antioxidant effect of these additives. Similar findings were observed in White leg shrimp supplied with CUR-NPs and in Nile tilapia supplemented with CUR-NPs [82, 108]. Recent investigations confirmed the positive role of CUR-NPs additions on the antioxidant status of Nile tilapia [2]. Various studies have recognized the beneficial impacts of *S. platensis* on the antioxidant ability of several fish species [45, 47, 52, 78, 110]. Curcumin’s antioxidant properties are linked with the activation of antioxidative enzymes and the nuclear transcription factor erythroid 2 (Nrf2) signaling pathways, which removes free radicals [2, 111]. Polyphenols found in these additives also promote antioxidant activity by scavenging ROS and preventing oxidative deterioration [112, 113]. Spirulina’s antioxidant properties can be attributed to its chemical composites, including vitamins, C-phycocyanins, β-carotene, and minerals, particularly phycocyanin, which alters cyclooxygenase-2 and guards against oxidative deterioration [47, 114].

The combination of CUR-NPs and SP diets revealed significant reductions in glucose, total cholesterol and triglyceride levels. The feeding diets of *Oreochromis mossambicus* supplied with curcumin also had significantly lower blood glucose concentrations [9]. Numerous reports have documented curcumin’s ability to promote glycogenesis and decrease blood glucose levels in Nile tilapia [25, 115]. Specifically, our study found that glucose, triglyceride, and total cholesterol levels have notably decreased in Nile tilapia upon feeding with various doses of CUR-NPs-supplemented diets [2]. Curcumin and *Spirulina platensis* have both been found to reduce triglyceride and total cholesterol in fish species such as tilapia, grass carp, and amberjack *Seriola dumerili* [116]. Also, we found that curcumin has a beneficial regulatory impact on lipid metabolism, decreasing the production of cholesterol and triglycerides in the liver and plasma [117, 118]. In addition, It has been discovered that Spirulina species’ polyphenol metabolites can lower body fat [119], which may be attributed to the decreased triglyceride and total cholesterol amounts in fish supplemented by *S. platensis* [78].

Furthermore, the outcomes of our data uncovered that the CUR-NPs and SP diets exhibited noticeable positive impacts on Nile tilapia biochemical blood metabolites associated with liver functions, including ALP, ALT, and AST, and indicators of renal tissue (uric acid and creatinine), showing that there is no liver failure or functional kidney impairment. Similar outcomes were confirmed in other studies involving curcumin in gilthead sea bream [83], and Nile tilapia given the CUR-NPs supplements [2]. Additionally, supplementing with *S. platensis* was found to enhance liver and renal functions in Nile tilapia [47].

Data showed that feeding *O. niloticus* with SP-diets and CUR-NPs led to a substantial rise in the total protein, albumin, and globulin levels in the fish’s serum. Similar conclusions were reported for rainbow trout fed with curcumin [28], as well as Nile tilapia supplemented with curcumin [115] and CUR-NPs [2]. *L. vannamei* fed with CUR-NPs-supplemented diets also exhibited higher albumin content as well as total protein levels [108], while Nile tilapia fed with live spirulina had higher levels of protein, albumin, and globulins [44]. Total serum proteins are an important indicator of healthy nutritional status for fish [19], and elevated levels of serum proteins and globulins are linked to strong innate immunity in fish species [120].

Many diseases and inescapable environmental pollutants endanger the well-being of fish [121]. Moreover, decreased disease resistance is another way that confinement stress can harm tissues and reduce productivity [122]. In our study, the control group showed tissue alterations in various fish organs, whereas the groups fed with CUR-NPs and SP exhibited fewer pronounced histological changes, highlighting the positive effect of nutrition in terms of disease resistance. Earlier studies have also shown the importance of diet in the occurrence and severity of various fish illnesses through the immune system’s regulation [123].

## Conclusions

The current research suggests that incorporating CUR-NPs or SP into the diet of Nile tilapia fingerlings can enhance the haemato-biochemical profile, redox status, performance and humoral immunity of fish. The study proved significant improvements in the efficiency of consumed feed, growth, immunity response, and antioxidant capacity. Additionally, there were no adverse impacts on the entire fish organs specifically, kidney and liver tissues. Finally, incorporating 5 g of SP or 30 mg CUR-Nps per kg diet is recommended to promote production sustainability and health of tilapia.

## Data Availability

All data generated and analyzed during this study are included in this published article.
